# Loss of Cytoplasmic CDK1 Predicts Poor Survival in Human Lung Cancer and Confers Chemotherapeutic Resistance

**DOI:** 10.1371/journal.pone.0023849

**Published:** 2011-08-24

**Authors:** Chunyu Zhang, Abdel G. Elkahloun, Matthew Robertson, Joell J. Gills, Junji Tsurutani, Joanna H. Shih, Junya Fukuoka, M. Christine Hollander, Curtis C. Harris, William D. Travis, Jin Jen, Phillip A. Dennis

**Affiliations:** 1 Medical Oncology Branch, Center for Cancer Research, National Cancer Institute, National Institutes of Health, Bethesda, Maryland, United States of America; 2 Cancer Genetics Branch, National Human Genome Research Institute, National Institutes of Health, Bethesda, Maryland, United States of America; 3 Medical Oncology Department, Kinki University School of Medicine, Osaka-Sayama, Osaka, Japan; 4 Biometric Research Branch, National Cancer Institute, National Institutes of Health, Bethesda, Maryland, United States of America; 5 Laboratory of Population Genetics, National Cancer Institute, National Institutes of Health, Bethesda, Maryland, United States of America; 6 Department of Surgical Pathology, Toyama University Hospital, Toyama, Japan; 7 Laboratory of Human Carcinogenesis, Center for Cancer Research, National Cancer Institute, National Institutes of Health, Bethesda, Maryland, United States of America; 8 Department of Pathology, Memorial Sloan-Kettering Cancer Center, New York, New York, United States of America; 9 Department of Pulmonary and Critical Care Medicine, Mayo Clinic, Rochester, Minnesota, United States of America; Istituto Dermopatico dell'Immacolata, Italy

## Abstract

The dismal lethality of lung cancer is due to late stage at diagnosis and inherent therapeutic resistance. The incorporation of targeted therapies has modestly improved clinical outcomes, but the identification of new targets could further improve clinical outcomes by guiding stratification of poor-risk early stage patients and individualizing therapeutic choices. We hypothesized that a sequential, combined microarray approach would be valuable to identify and validate new targets in lung cancer. We profiled gene expression signatures during lung epithelial cell immortalization and transformation, and showed that genes involved in mitosis were progressively enhanced in carcinogenesis. 28 genes were validated by immunoblotting and 4 genes were further evaluated in non-small cell lung cancer tissue microarrays. Although CDK1 was highly expressed in tumor tissues, its loss from the cytoplasm unexpectedly predicted poor survival and conferred resistance to chemotherapy in multiple cell lines, especially microtubule-directed agents. An analysis of expression of CDK1 and CDK1-associated genes in the NCI60 cell line database confirmed the broad association of these genes with chemotherapeutic responsiveness. These results have implications for personalizing lung cancer therapy and highlight the potential of combined approaches for biomarker discovery.

## Introduction

Lung cancer is the leading cause of cancer death throughout the world, causing approximately 1.2 million deaths annually and an estimated 157,300 deaths in the US in 2010 [1]. NSCLC most commonly occurs in smokers [2]. In the US, approximately 90% of deaths from lung cancer in men and 79% in women are associated with smoking [3]. Most lung cancers are of epithelial cell origin. Therefore, the development of lung cancer reflects the cumulative effect of smoking-induced molecular changes in airway epithelial cells. Because approximately 90,000,000 current or former smokers in the US have a permanently increased relative risk for developing lung cancer, the identification of early molecular events inherent to lung tumorigenesis could provide a basis for interventions aimed at preventing the phenotypic progression that underlies carcinogenesis.

Molecular changes in lung cancer are complex and include genetic, epigenetic, and biochemical alterations. The first molecular events to be identified were genetic and included inactivation of tumor suppressor genes such as p53 [4], and activation of oncogenes such as K-Ras [5]. Epigenetic events include silencing of the CDK inhibitor, p16, the tumor suppressor RASSF1, and other genes through methylation [6], [7]. Recently, biochemical events such as constitutive activation of signal transduction pathways that promote cellular survival and therapeutics resistance have also been reported [8], [9], [10]. Ultimately, these molecular changes must drive phenotypic progression of the epithelial cell destined for transformation. The experimental transformation of normal epithelial cells into tumorigenic cells requires two distinct steps. The first is cell immortalization, which is required to make cells susceptible to the second step, transformation. Human cells must circumvent two barriers to achieve immortalization, replicative senescence and cellular crisis. These barriers to immortalization are regulated by telomere shortening and by the retinoblastoma (Rb) and p53 tumor-suppressor pathways [11]. Additional changes are required for full transformation. Even though an increasing number of molecular changes have been identified in the phenotypic progression of epithelial cell transformation, other events are likely important.

To identify a broad range of molecular changes inherent to the processes of immortalization and transformation of airway epithelial cells, we used a model system established by Reddel *et al.*, who created immortalized, non-tumorigenic bronchial epithelial cells (BEAS-2B) by infecting normal human bronchial epithelial cells (NHBE) with adenovirus-12 SV40 hybrid virus [12]. Disruption of p53 and Rb pathways by SV40 T-antigen, together with increased telomerase expression, immortalized BEAS-2B cells. BEAS-2B cells were then made fully tumorigenic by exposing BEAS-2B cells in vivo to the tobacco specific carcinogen, nitrosamine 4-(methylnitrosamino)-1-(3-pyridyl)-1-butanone (NNK), for 6 months. NNK exposure induced phenotypic changes in BEAS-2B cells that were similar to the progressive changes that occur during human lung carcinogenesis [13].

**Figure 1:**
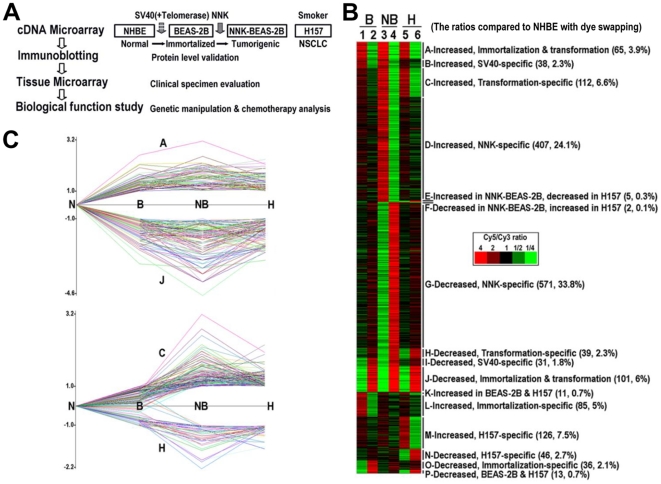
Classification and annotation of differentially expressed genes in BEAS-2B, NNK-BEAS-2B and H157 compared to NHBE. A. The system and strategy to study gene expression profile, its clinical significance and functional role during NSCLC carcinogenesis and chemotherapeutic resistance. B. Classification of 1,688 regulated genes among BEAS-2B (B), NNK-BEAS-2B (NB) and H157 (H) compared to NHBE (N). Genes whose expression level did not change between cell lines were excluded. The ratios graphically visualized by Cluster and TreeView programs (http://rana.lbl.gov/EisenSoftware.htm). Pairs of lanes and reciprocal red and green colors indicate dye swapping. C. Gene expression dynamics during immortalization and/or transformation. The graphs were generated by Cluster Analysis of Gene Expression Dynamics program (http://www.genomethods.org/caged). A, C, J and H symbols depict groups of genes identified in Figure 1B.

In these studies, we systematically profiled gene expression in normal (NHBE), immortalized (BEAS-2B) and fully transformed (NNK-BEAS-2B) human bronchial epithelial cells, as well as a non-small cell lung cancer (NSCLC) cell line (H157) from a smoker (Figure 1A). Expression profiles that accompany the immortalization and/or transformation of bronchial epithelial cells were generated, and expression of 28 genes was validated by immunoblotting. 4 of them were further evaluated in immunohistochemical analyses of tissue microarrays that contain NSCLC specimens, surrounding non-diseased tissues and non-pulmonary normal tissues. Although all 4 genes were predominantly expressed in tumor tissues, loss of expression of cytoplasmic CDK1 was clinically important because it was associated with a poor prognosis for NSCLC patients. This poor prognostic value may be associated with therapeutic resistance, because decreasing levels of cytoplasmic CDK1 *in vitro* increased resistance to standard chemotherapies used in the treatment of NSCLC, especially microtubule agents where resistance was almost complete. These studies illustrate how a combined microarray approach can facilitate the identification of new, relevant targets in cancer.

## Results

### Classification of differentially expressed genes in BEAS-2B, NNK-BEAS-2B and H157 cells compared to NHBE cells

With NHBE as the reference, 1,688 genes were differentially expressed in BEAS-2B, NNK-BEAS-2B and/or H157 cells. Differentially expressed genes were classified into lettered groups (A-P) based on the distribution of the expression changes (Figure 1B). Gene expression profiles were grouped based on phenotypic characteristics. NHBE, BEAS-2B, NNK-BEAS-2B cells represented normal, immortalized, and transformed stages of lung carcinogenesis, respectively, and H157 cells were used as an unrelated fully transformed cell line derived from a NSCLC patient. Genes in groups A (65, 3.9%) or J (101, 6%) were upregulated or downregulated, respectively, in BEAS-2B, NNK-BEAS-2B and H157 cells, indicating that these genes expression changes were common to the immortalization and transformation of NHBE. Expression of genes in groups B (38, 2.3%) or I (31, 1.8%) was increased or decreased, respectively, in BEAS-2B and NNK-BEAS-2B but not H157 cells, suggesting that these changes resulted from SV40-mediated immortalization. Genes in groups C (112, 6.6%) or H (39, 2.3%) were differentially expressed in NNK-BEAS-2B and H157 cells, implicating that these genes contribute to full cell transformation. Groups D (407, 24.1%) and G (571, 33.8%) comprised the largest number of genes and were specific for NNK-BEAS-2B cells. This suggests that NNK-mediated transformation is a complex event that requires changes in expression of many genes. Other changes in gene expression were specific for a given cell type, and were excluded from further analysis. The complete gene list and related ratio data are provided in supporting information (Table S1).

### Gene expression profile annotation and biological analysis

Among the classifications, groups A and J include genes that are crucial throughout tumor evolution, from immortalization to transformation, and groups C and H include genes that are associated with full transformation. The quantitative changes in gene expression are shown (Figure 1C). To analyze gene expression changes of these groups on a genomic scale and provide linkage to putative biological processes, we performed High-Throughtput GoMiner analysis (Table 1, the related genes were listed in Table S2). In group A, genes involved in mitosis were highly enriched. In group J, genes involved in ectoderm development were diminished. No significant gene ontology (GO) categories were noted for groups B and I. In groups C or H where gene expression changes were associated with transformation, genes involved in mitosis regulation were enhanced, while genes that negatively regulate cell proliferation and biological processes were diminished (Table 1). Several GO categories were noted for groups D and G that are related to changes associated with transformation by NNK (Table S3). These results were extended by performing Gene MicroArray Pathway Profiler analysis, which showed that the cumulative effect of gene expression changes in carcinogenesis would be predicted to increase autonomous progression through the cell cycle (Figure S1) and evasion of apoptosis (Figure S2).

**Table 1:** GeneOntology analysis of gene expression signatures during immortalization and/or transformation.

GO category	Total	Changed	Enrichment	Log10(p)	FDR
**A**					
GO:0007067_mitosis	119	10	14.23	−9.00	0.00
GO:0000910_cytokinesis	121	10	14.00	−8.93	0.00
GO:0000278_mitotic_cell_cycle	175	11	10.65	−8.54	0.00
GO:0007049_cell_cycle	486	15	5.23	−7.41	0.00
GO:0000067_DNA_replication_and_chromosome_cycle	9	4	75.25	−6.89	0.00
GO:0006259_DNA_metabolism	349	11	5.34	−5.47	0.00
GO:0007059_chromosome segregation	40	4	16.94	−4.09	0.00
GO:0007088_regulation of mitosis	26	3	19.54	−3.35	0.00
GO:0006260_DNA_replication	113	5	7.49	−3.30	0.00
GO:0000075_cell_cycle_checkpoint	27	3	18.82	−3.30	0.00
GO:0000070_mitotic_sister_chromatid_segregation	31	3	16.39	−3.12	0.00
**J**					
GO:0007398_ectoderm_development	50	11	21.06	−11.72	0.00
GO:0008544_epidermis_development	42	9	20.52	−9.53	0.00
GO:0009888_histogenesis	94	11	11.20	−8.61	0.00
GO:0009887_organogenesis	664	20	2.88	−5.08	0.00
GO:0009628_response_to_abiotic_stimulus	264	10	3.63	−3.43	0.00
**C**					
GO:0007088_regulation_of_mitosis	26	4	14.31	−3.81	0.02
GO:0000279_M_phase	148	8	5.03	−3.77	0.01
GO:0007067_mitosis	119	7	5.47	−3.58	0.01
GO:0000278_mitotic_cell_cycle	175	8	4.25	−3.28	0.01
GO:0000398_nuclear_mRNA_splicing_via_spliceosome	70	5	6.65	−3.06	0.01
**H**					
GO:0008285_negative_regulation_of_cell_proliferation	108	4	9.06	−3.05	0.05

GeneOntology analysis was performed using High-Throughput GoMiner. A, C, J and H symbols depict groups of genes identified in Figure 1B.

### Validation of expression profiles at protein level by immunoblotting

**Figure 2:**
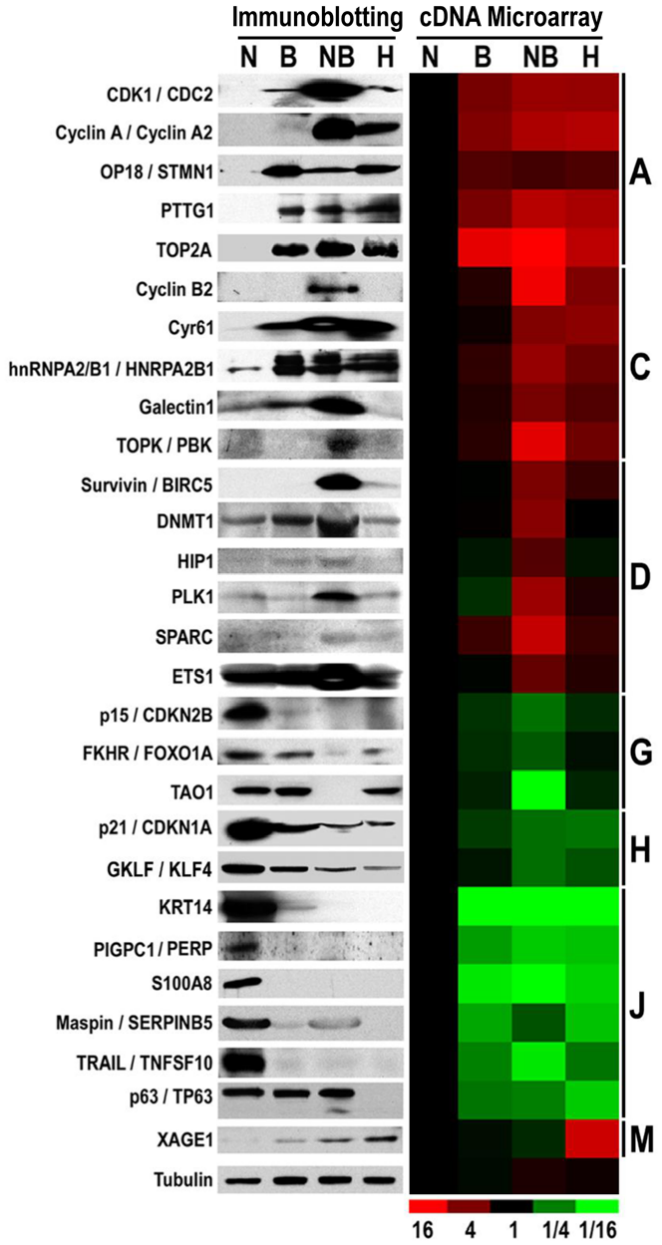
Immunoblotting and comparison of expression changes between protein and mRNA levels. Immunoblotting analysis in NHBE (N), BEAS-2B (B), NNK-BEAS-2B (NB) and H157 (H) cells for 28 Molecules from representative groups, and the comparison of expression changes with mRNA levels from microarray data.

To validate our microarray data, we performed immunoblotting for 28 candidate genes drawn from various groups (Figure 2). The choice of genes was based on the commercial availability of antibodies that had been previously used in immunoblotting experiments. Groups A or J represented genes that were over- or under-expressed in immortalization and transformation, respectively. Compared to NHBE, BEAS2B, NNK-BEAS-2B and H157 cells had increased expression of CDK1, Cyclin A, STMN1, PTTG1, and TOP2A (group A), and decreased expression of KRT14, PERP, S100A8, Maspin, TRAIL, and p63 (group J). When comparing mRNA expression to protein expression in groups A and J, only p63 appeared misclassified in that p63 was categorized into group J based on microarray data, but immunoblotting revealed that decreased p63 expression was only observed in H157 cells (group N). This could be related to variable antibody-specific reactivity against p63 isoforms and splice variants. The genes in groups C or H were classified as transformation-specific based on microarray data. Immunoblotting revealed that many genes within this group C were misclassified. For example, Cyclin B2, Galectin1 and PBK appeared to be predominantly expressed in NNK-BEAS-2B cells (group D), while Cyr61 and hnRNPA2/B1 were common to cells that were immortalized and transformed (group A). p21 and KLF4 from group H were accurately classified, because their mRNA and protein levels were decreased in NNK-BEAS-2B and H157 transformed cells. Groups D or G represented NNK-specific genes. NNK-BEAS-2B cells uniquely upregulated protein expression of Survivin, DNMT1, HIP1, PLK1, SPARC and ETS1 (group D), and downregulated protein expression of p15, FKHR and TAO1 (group G). The correlation between mRNA expression and protein expression was high for these groups. XAGE1, originally classified as an H157 cell-specific gene (group M), showed greatest expression in H157 cells. Overall, immunoblotting revealed that 22/28 genes (79%) were correctly classified from the microarray data, indicating a high correlation between mRNA and protein expression.

### Immunohistochemical staining on lung tissue microarray

**Figure 3:**
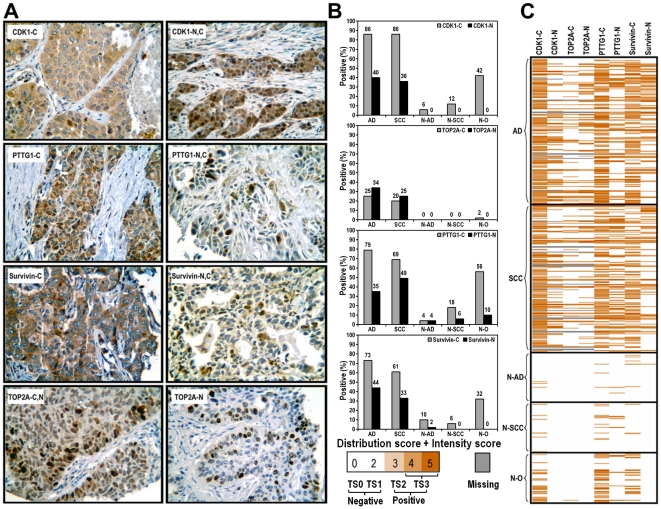
Tissue microarray immunohistochemistry. A. Examples of CDK1, PTTG1, Survivin and TOP2A subcellular staining in clinical tumor samples (-C: cytoplasmic staining; -N: nuclear staining). Staining is shown at 400× magnification. B. Tissue distribution of positive rates for CDK1, PTTG1, Survivin and TOP2A. AD: Adenocarcinoma; SCC: Squamous cell carcinoma; N-AD: Adjacent non-diseased lung tissues from Adenocarcinoma; N-SCC: Adjacent non-diseased lung tissues from squamous cell carcinoma; N-O: Non-pulmonary normal organs. C. Overview of entire tissue microarray staining for expression of these antigens in the cytoplasm or nucleus.

To determine whether genes that were identified in the microarray analysis and further validated using immunoblotting had clinical significance, we performed immunohistochemistry (IHC) to assess expression in human NSCLC specimens. Of the 22 correctly classified genes, CDK1, TOP2A, PTTG1 and Survivin were chosen based on commercial availability of antibodies that had been validated in IHC. Expression was assessed in tissue microarrays (TMAs) that contain 300 NSCLC cases (150 adenocarcinomas (AD) and 150 squamous cell carcinomas (SCC)), 100 adjacent non-diseased lung tissues from AD and SCC cases, and 50 non-pulmonary normal tissues [14]. Scoring was based on intensity, distribution, and subcellular localization. Staining patterns for CDK1, PTTG1 and Survivin were similar in that staining was either predominantly cytoplasmic or both nuclear and cytoplasmic. In contrast, staining for TOP2A was either exclusively nuclear or both cytoplasmic and nuclear (Figure 3A). Compared to tumor cells, staining of surrounding stromal tissue was minimal or absent.

All four proteins were predominantly expressed in tumor tissues, compared to the adjacent non-diseased lung tissues (Figure 3B). This was especially evident when nuclear staining was considered. Most NSCLC samples were positive for CDK1 cytoplasmic staining (86% for AD and SCC), and 40% of AD specimens and 36% of SCC specimens showed nuclear staining. Surrounding normal lung tissues and other normal tissues expressed cytoplasmic CDK1, but did not express nuclear CDK1. Staining for TOP2A was overall less prevalent in NSCLC specimens than for the other proteins (20–34%), but was more evenly distributed between cytoplasmic and nuclear staining. Staining for TOP2A was exclusive to NSCLC specimens vs. surrounding normal lung tissues. PTTG1 and Survivin showed a high prevalence of cytoplasmic staining (PTTG1- 79% in AD and 69% in SCC; Survivin- 73% in AD and 61% in SCC), as well as nuclear staining (PTTG1- 35% in AD and 49% in SCC; Survivin- 44% in AD and 33% in SCC). Overall, staining for all four proteins was selective for NSCLC specimens vs. surrounding normal tissues, regardless of subcellular localization (Figure 3C).

**Figure 4:**
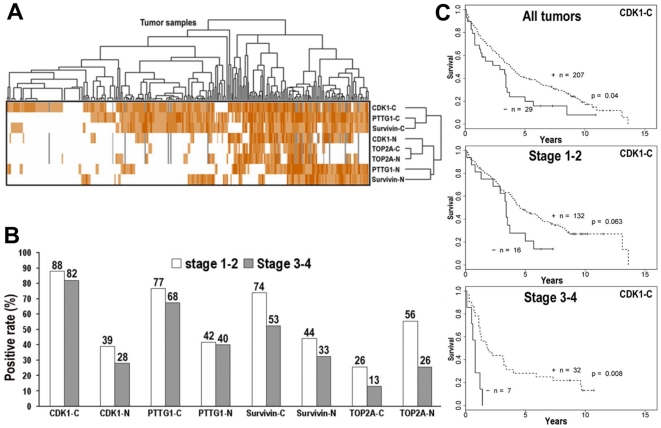
The associations of staining outcomes with tumor samples, clinical factors and patient survival. A. The hierarchial clustering for both antibodies and tumors using Cluster 3.0 and Java TreeView. B. The positive rates of CDK1, PTTG1, Survivin and TOP2A in early stages (1–2) and advanced stages (3–4). C. Kaplan-Meier survival analysis for cytoplasmic CDK1 staining pattern in NSCLC. The permutation test was used to calculate the *p* value for all tumors and tumors by stage.

We used two-way clustering to analyze the staining patterns, and the χ^2^ test to examine the agreement between each antibody and the association of antibody staining with clinical factors. Tumors that stained for TOP2A were most distinct, compared to CDK1, PTTG1 and Survivin (Figure 4A). The strongest association of antibody staining was between TOP2A nuclear and cytoplasmic staining (Table 2), which is consistent with the observation that TOP2A is localized to the nucleus and often accompanied by cytoplasmic staining (Figure 3A). In contrast to TOP2A, staining for CDK1, PTTG1, and Survivin was predominantly cytoplasmic. Cytoplasmic or nuclear staining for PTTG1 and Survivin also clustered very similarly, and this was consistent with the χ^2^ tests (Table 2). CDK1 nuclear staining was strongly associated with either nuclear or cytoplasmic TOP2A staining. Overall, the agreement between staining with these antibodies was very strong, except for the association of cytoplasmic CDK1 staining with nuclear Survivin staining (Figure 4A and Table 2).

**Table 2:** Associations of IHC staining, clinical factors and patient survival.

IHC	CDK1-N	CDK1-C	PTTG1-N	PTTG1-C	Survivin-N	Survivin-C	TOP2A-N	TOP2A-C
CDK1-N		6.89E-05	7.60E-10	5.10E-09	1.64E-05	4.33E-05	8.18E-12	7.81E-14
CDK1-C			0.00336	0.000187	–	9.62E-07	3.07E-05	0.000182
PTTG1-N				9.46E-09	2.70E-08	3.47E-05	6.75E-07	1.02E-06
PTTG1-C					3.11E-07	1.48E-22	6.75E-07	1.02E-06
Survivin-N						3.02E-07	0.000376	0.008537
Survivin-C							2.93E-05	2.84E-05
TOP2A-N								2.29E-24
TOP2A-C								
**Clinical**								
age	–	–	–	–	–	–	–	–
gender	–	–	–	–	–	–	–	–
differentiation	–	–	–	–	–	–	–	–
size	–	–	–	–	–	–	–	0.00745^a^
stage	–	–	–	–	–	0.0436^b^	–	–
**Survival**								
all histology	–	–	–	–	–	–	–	–
AD	–	–	–	–	–	–	–	–
SCC	–	–	–	–	–	–	–	–
<5 cm	–	–	–	–	–	–	–	–
> = 5 cm	–	–	–	–	–	–	–	–
stage 1–2	–	–	–	–	–	–	–	–
stage 3–4	–	0.008^c^	–	–	–	–	–	–
overall	–	0.04^d^	–	–	–	–	–	–

a Cytoplasmic TOP2A staining was strongly associated with smaller tumors (<5 cm).

b Cytoplasmic Survivin staining was associated with stage I disease (stage 1>stage 2-3-4).

c Loss of cytoplasmic CDK1 staining at stage 3–4 was strongly associated with poor survival.

d Loss of cytoplasmic CDK1 staining was associated with poor survival.

When analyzed by stage, staining for all four proteins was greater in stage 1–2 disease (Figure 4B). This was especially true for TOP2A nuclear staining, where 56% of stage 1–2 specimens were positive whereas only 26% of stage 3–4 specimens were positive. When staining was associated with clinical variables, there was no relationship with age, gender or differentiation (Table 2). Cytoplasmic TOP2A staining was strongly associated with smaller tumors (<5 cm) (*p* = 0.00745), and cytoplasmic Survivin staining was associated with stage I disease (stage 1>stage 2-3-4, *p* = 0.0436) (Table 2).

Only cytoplasmic CDK1 staining was associated with survival, although there was a trend toward worse survival with cytoplasmic PTTG1 positive patients with tumors <5 cm (*p* = 0.089). Regardless of stage, loss of cytoplasmic CDK1 confers a poor prognosis (*p* = 0.040) (Table 2). Since the number of stage 2 tumors negative for cytoplasmic CDK1 is too small and prohibited a meaningful comparison with positive stage 2 tumors, and the overall survival of patients in this data set with stage 1 and stage 2 patients is similar, we combined stage 1 and stage 2 tumors as early stage tumors. When comparing early stage (1 and 2) and advanced stage tumors (3 and 4), we found that loss of cytoplasmic CDK1 was borderline significant for early stage tumors (*p* = 0.063) but highly significant for late stage tumors (*p* = 0.008) (Figure 4C and Table 2).

### Loss of cytoplasmic CDK1 and chemotherapy resistance

**Figure 5:**
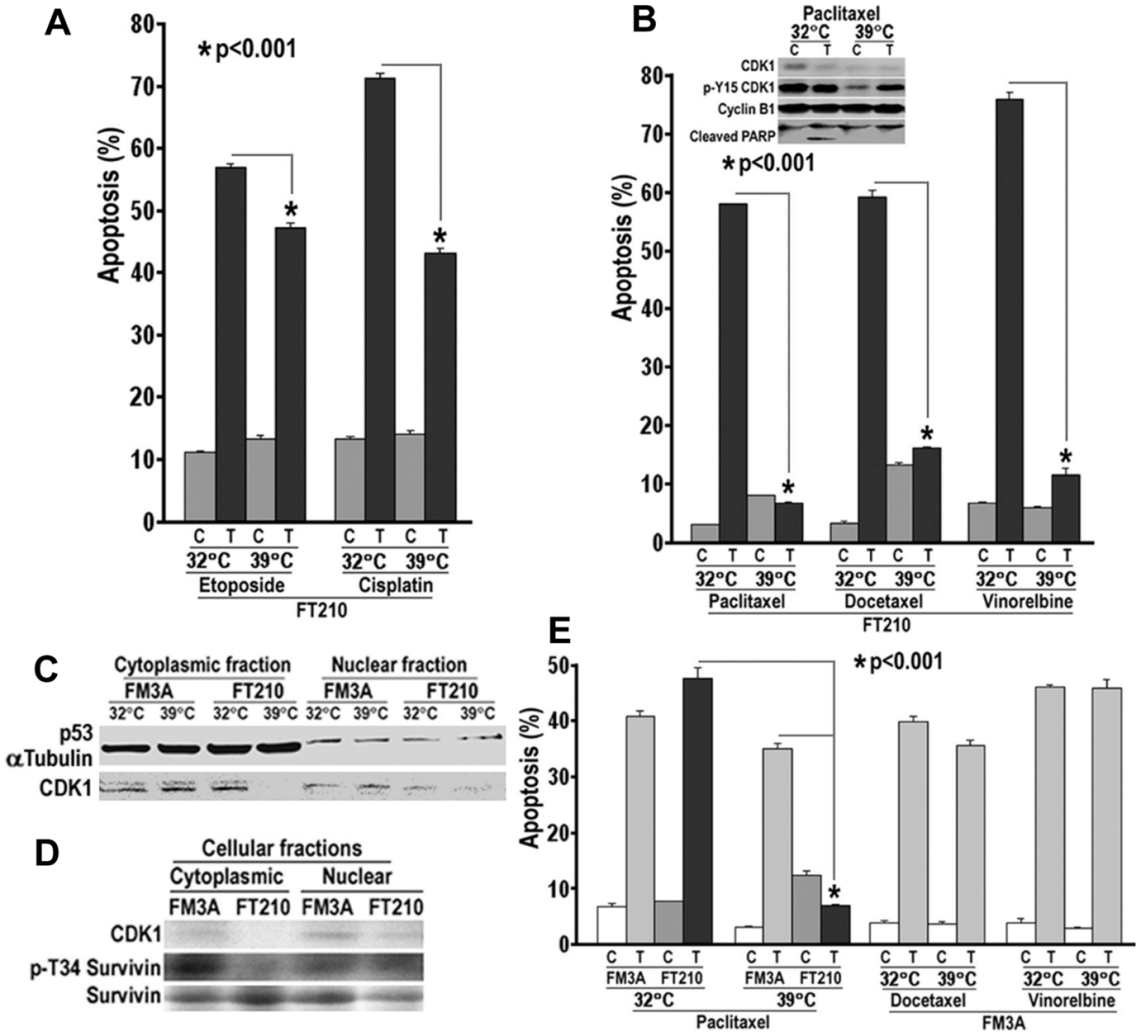
Loss of CDK1 protein and activity and chemotherapeutic resistance. A. FT210 cells were serum-starvated in 0.1% FBS RPMI1640 medium overnight, then treated with 10 µM etoposide or cisplatin in 0.1% FBS containing medium for 4 days at 32°C or 39°C. Apoptosis (sub-G1 DNA content) was determined by flow cytometry (C: Control; T: Treatment). B. FT210 cells were cultured in normal medium and treated with 1 µM paclitaxel or docetaxel and 0.1 µM vinorelbine for 1–4 Days. The CDK1 protein was detected at Day 3 using CDK1 or p-Y15 CDK1 antibodies, and apoptotic death was monitored by PARP cleavage. Apoptosis was determined at Day 4. C. FT210 and its parental cell line FM3A cells were cultured for 6 h then subjected to subcellular fractionation for immunoblotting analysis. αTubulin and p53 served as loading controls for cytoplasmic and nuclear fractions, respectively. D. FM3A and FT210 cells were cultured at 39°C for 72 h and subjected to subcellular fractionation. Expression of CDK1, p-Survivin (Thr34) and Survivin was assessed by immunoblotting. E. FM3A and FT210 cells were treated with 1 µM paclitaxel or docetaxel and 0.1 µM vinorelbine. Apoptosis was determined at Day 4.

The fact that loss of cytoplasmic CDK1 conferred a poor prognosis, especially for advanced NSCLC patients who are more likely to receive chemotherapy or chemoradiotherapy, suggested it might contribute to chemotherapeutic resistance. To test this hypothesis, a temperature-sensitive p34CDC2/CDK1 mutant murine carcinoma FT210 cell line was used. When FT210 cells are cultured at a non-permissive temperature of 39°C from a permissive temperature of 32°C, CDK1 becomes preferentially inactivated and degraded from the cytoplasm [15]. Chemotherapies typically used to treat NSCLC were tested in these cells at each temperature. Etoposide, a topoisomerase II inhibitor, or cisplatin, a DNA damaging agent, significantly increased apoptosis at the permissive temperature. At the non-permissive temperature, apoptosis was partially inhibited (Figure 5A). Similar results were observed at the permissive temperature for three different microtubule-based therapies, where marked increases in apoptosis were observed (Figure 5B). In contrast, under non-permissive conditions, cells were almost completely resistant to each microtubule-targeted agent. Total levels of CDK1 protein were decreased at 39°C, as was phosphorylation of CDK1 at Y15. Phospho-Tyr15 CDK1 is an inactive cytoplasmic form of CDK1 due to phosphorylation by Myt1. Cleavage of PARP at each temperature by paclitaxel was consistent with measurement of apoptosis (Figure 5B). Compared to its parental cell line FM3A, FT210 cells preferentially lost cytoplasmic CDK1 protein expression (Figure 5C) and activity indicated by decreased phosphorylation of survivin at Thr34, a site for CyclinB-CDK1 kinase [16] (Figure 5D). Paclitaxel caused significant cytotoxicity in FM3A and FT210 cells at 32°C, but only FM3A cells at 39°C. Similarly, FM3A cells were killed by docetaxel and vinorelbine at either 32°C or 39°C (Figure 5E). Thus, loss of CDK1 in FT210 cells increased relative resistance to etoposide and cisplatin, but induced marked resistance to microtubule targeting agents.

**Figure 6:**
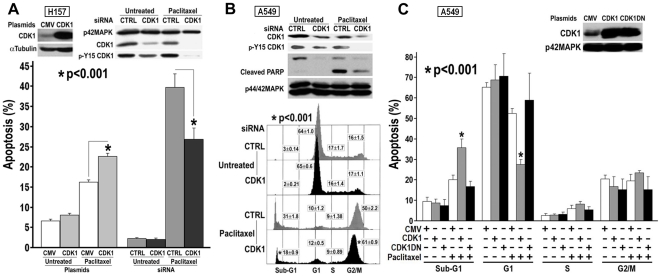
Modulation of CDK1 protein level and activity and sensitivity of NSCLC cells to Paclitaxel treatment. A. CDK1 protein over or under expressed in H157 cells. Cells were transfected with *CDK1* plasmid or siRNA, 48 h or 72 h later, were treated with 1 µM paclitaxel for 2 days. CDK1 protein level was monitored by CDK1 or p-Y15 CDK1 antibodies. Sub-G1 DNA content was determined by flow cytometry. B. Knockdown of CDK1 in A549 cells. Cells were transfected with *CDK1* siRNA, 72 h later, treated with 1 µM paclitaxel for 2 days. Apoptosis was determined by PARP cleavage using immunoblotting or sub-G1 DNA content using flow cytometry. CDK1 protein level was monitored by CDK1 or p-Y15 CDK1 antibodies. C. Overexpression of CDK1 or CDK1DN in A549 cells. Cells were transfected with *CDK1* or *CDK1DN* plasmids, 48 h later, cell cycling stopped by overnight pretreatment of low serum (0.1% FBS) medium with PI3K inhibitor LY294002 (10 µM), then cells were treated with 1 µM paclitaxel in low serum medium containing LY294002 (10 µM, freshly added) for 2 days. Sub-G1 fraction and cell cycle profile were determined by flow cytometry. Over expression of CDK1 and CDK1DN was detected by CDK1 antibody.

We further investigated the role of CDK1 in chemotherapeutic resistance by modulating levels of CDK1 in human NSCLC cells. Overexpression of CDK1 in H157 cells slightly elevated basal levels of apoptosis and potentiated paclitaxel-induced apoptosis (Figure 6A). In contrast, downregulation of CDK1 in H157 cells using siRNA conferred resistance to paclitaxel. Similar results were observed in a separate NSCLC cell line (A549) when CDK1 expression was decreased using siRNA (Figure 6B). Although knockdown of CDK1 did not affect cell cycle distribution of untreated A549 cells, it did reduce sub-G1 DNA content and PARP cleavage when A549 cells were exposed to paclitaxel. The increase in G2/M fraction with CDK1 knockdown is consistent with the role of CDK1 at the G2 to M phase transition. To dissect a role for CDK1 in apoptosis vs. G2/M transition, A549 cells were transfected with empty vector, wild type *CDK1* or dominant negative *CDK1* (*CDK1DN*) constructs, growth arrested with serum deprivation and a PI3K inhibitor LY294002, and then treated with paclitaxel. Diminished cell proliferation was observed microscopically and confirmed by the lack of G2/M enrichment with paclitaxel treatment (Figure 6C). Despite cell cycle arrest, overexpression of CDK1 still increased the sub-G1 fraction that correlated with loss of the G1 population. These effects were not seen with overexpression of CDK1DN, which lacks kinase activity via a dominant point mutation (D146N) [17].

**Figure 7:**
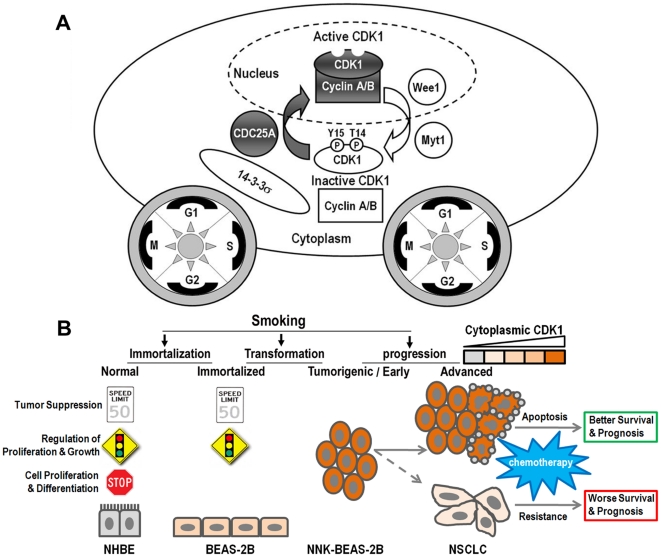
Dual role of CDK1 in tumorigenesis and chemotherapeutic sensitivity. A. A model of CDK1 activation. When inactive p-Y15/T14 CDK1 is dephosphorylated on Y15 and T14 by CDC25A, it associates with Cyclin A/B and becomes active within the cytoplasm, then quickly translocates into the nucleus, where it promotes G1 to S and G2/M transitions. Active CDK1 is inactivated via sequential phosphorylation of Y15 and T14 by Wee1 and Myt1, and sequestrated by 14-3-3σ in the cytoplasm. This process repeats with cell cycling to drive cellular proliferation. B. A diagram to illustrate the dual role of CDK1 as a tumorigenic driver in lung carcinogenesis and a sensitizer in chemotherapeutic responsiveness.

The above experiments demonstrate that CDK1 protein level and activity is associated with the sensitivity of cancer cells to chemotherapy. To confirm this observation, we searched the NCI60 human cancer cell line compound sensitivity and molecular targets databases and performed COMPARE [18] analyses. Expression of the CDK1 activator CDC25A, p-Y15/T14 CDK1, total CDK1, as well as the CDK1 binding partners cyclin B and cyclin A were positively associated with sensitivity to paclitaxel, etoposide, and docetaxel. The highest Pearson Correlation Coefficients (PCC) were observed with docetaxel and paclitaxel (Table 3). Although CDC25A, p-Y15 CDK1 and cyclin A were modestly associated with cisplatin, CDK6 and TYMS were the top correlates. Thus, CDK1 and its activating machinery components (Figure 7A) were broadly identified as factors that underlie sensitivity to many agents used to treat human cancers, especially microtubule-directed agents.

**Table 3:** Correlation between CDK1 activating machinery components and chemotherapeutic agents in NCI60 cell line panel.

PCC (Ranked if PCC≥0.200)			CDC25A	p-Y15 CDK1	p-T14 CDK1	CDK1	Cyclin B	Cyclin A
			MT1159	MT1161	MT1160	MT1158	MT1222	MT1167
Paclitaxel	NSC125973	−4.6 M	0.451 (1)	0.282 (3)	0.214 (6)	0.159	0.318 (2)	0.267 (4)
Docetaxel	NSC628503	−7.0 M	0.553 (1)	0.495 (2)	0.297 (5)	0.247 (6)	0.466 (3)	0.357 (4)
Etoposide	NSC141540	−3.0 M	0.394 (1)	0.259 (5)	0.036	0.148	0.136	0.202 (6)
Cisplatin	NSC119875	−4.3 M	0.134	0.167	−0.060	−0.096	−0.105	0.285 (3)

A COMPARE analysis was performed by correlating protein levels of CDK1 activating machinery components with GI_50_ (concentration producing 50% growth inhibition) values of chemotherapeutic agents in NCI60 human cancer cell lines. A positive Pearson Correlation Coefficient (PCC) indicates that the greater abundance of the target may associate with sensitivity to the drug, whereas a negative correlation is indicative of more target conferring cellular resistance to the given drug.

## Discussion

The identification of clinically relevant protein targets in cancer is difficult. We strategically profiled transcriptional alterations in models of lung carcinogenesis, validated targets at a protein level, and evaluated expression and subcellular localization of the top candidates on clinical tissue microarrays. Of the genes identified in cDNA microarrays and corroborated by immunoblotting, CDK1, TOP2A, PTTG1 and Survivin were chosen for further evaluation. CDK1, PTTG1 and Survivin were highly expressed in lung tumor tissues. Nuclear CDK1 and TOP2A was exclusively expressed in tumor tissues compared to normal tissues, including non-pulmonary normal organs. The fact that these genes were preferentially expressed in tumor tissues, especially in early stage NSCLC, suggests that they might have utility as biomarkers and therapeutic targets in early stage NSCLC.

CDK1 is an important, emerging target in cancer. CDK1 is a catalytic subunit of M-phase promoting factor, which is essential for G1/S and G2/M phase transitions of the eukaryotic cell cycle. The highest staining in NSCLC clinical samples and the strongest expression in tumorigenic NNK-BEAS2B cells suggest that CDK1 plays a pivotal role in tumorigenesis. In fact, overexpression of CDK1 has been observed in early stage lung adenocarcinomas [19]. Our data showed that although most NSCLC specimens expressed cytoplasmic CDK1, its loss conferred a poor prognosis, especially for advanced NSCLC patients. Loss of cytoplasmic CDK1 could be explained by loss of total protein expression or translocation to the nucleus. Very few tumor samples (4 cases) express nuclear but not cytoplasmic CDK1, suggesting that loss of proteins such as 14-3-3σ that sequester CDK1 in the cytoplasm might contribute to this finding [20] [21], but loss or reduction of 14-3-3σ is very rare in primary NSCLC specimens [4]. Our chemotherapy experiments demonstrate that lowering total levels of CDK1 or cytoplasmic levels of CDK1 confers chemotherapeutic resistance to many agents used to treat lung cancer, especially microtubule-directed agents.

Data derived from the NCI 60 human cancer cell line database suggests that the status of many components of the CDK1 machinery could potentially play a broad role in sensitivity to chemotherapy that is not only limited to NSCLC cell lines. CDC25 dual-specificity phosphatases are key regulators of cell cycle progression through activation of CDKs by dephosphorylating CDKs at threonine 14 and tyrosine 15, two critical amino acid residues leading to subsequent association of CDKs with their associated cyclins. Three homologs exist in mammals: CDC25A, CDC25B, and CDC25C. CDC25A [22], [23], [24] and CDC25B have oncogenic properties and are overexpressed in some types of tumors [25]. CDC25B and CDC25C are implicated as regulators of mitosis but loss of these genes in mice or cells does not alter cell cycle or checkpoint responses, suggesting a possible functional compensation by CDC25A [26]. The CDC25A protein shuttles between the nucleus and cytoplasm [27], [28] and has a comprehensive function in regulating G1/S, S and G2/M transitions [29]. Thus, CDC25A temporally and spatially has more chances than CDC25B/C to prematurely activate CDK1. Accompanying CDK1 dephosphorylation by CDC25A, CDK1 associates with cyclin A/B and then moves rapidly into the nucleus [30]. The cell cycle has to be regulated in both time and space [31]. An unscheduled, premature or sustained CDK1 activation can be lead to cell death, mitotic catastrophe or apoptosis induction [32], [33], [34], especially when CDK1 activating machinery components are highly expressed [35]. Phosphorylated CDK1, an inactive form predominantly in cytoplasm [31], is a substrate for CDC25A, and its abundance in the cytoplasm enhances the CDK1 activation potential. This might be the reason why p-Y15/T14 CDK1 was more positively associated drug sensitivity than total CDK1 in the COMPARE analysis, and why cytoplasmic CDK1 loss confers chemotherapeutic resistance and worse survival in NSCLC patients.

Our High-Throughput GoMiner analysis revealed that mitosis is the major biological process that is altered during NSCLC carcinogenesis. Mitotic CDK1 is the only essential cell cycle CDK to promote unlimited cell division and a key player in oncogenic growth [36]. However, high level of CDK1 activation might sensitize tumor cells to stresses either from nutrient depletion or chemotherapy treatment. Because deficiency in Cdk1 results in no cell division [37], CDK1 loss from cancer cells is unlikely a genetic or early event in tumorigenesis. With tumor progression, the original requirement for high level of CDK1 activation may not be necessary when more favorable genetic and epigenetic changes occur. Under selective pressure for survival, cancer cells with low CDK1 activation might acquire advantages for expansion and metastasis (Figure 7B). This is supported by recent observations, such as Cdk1 activity impaired by the absence of Fez1/Lzts1 predisposes mice to cancer development [38], inhibition of Cdk1 mediates protection from apoptosis in Cdc20-depleted cells [39], a low ratio of CDK2 to CDK1 activity is associated with a favorable prognosis in breast cancer patients [40], phosphorylation of p62 by Cdk1 is necessary to restrain tumorigenesis *in vivo* in response to Ras-induced transformation [41], and CDK1 inhibits cancer cell migration and invasion by phosphorylation of EZH2 at Thr 487 [42].

Although CDK1 is perhaps the most interesting target identified in our studies, other data also supports targeting TOP2A, PTTG1 and Survivin. For example, TOP2A gene expression is regulated by p53 gene status in NSCLC patients, and its overexpression induced by mutant p53 might increase tumor aggressiveness [43]. Identification of TOP2A in our studies is consistent with the fact that an inhibitor of topoisomerase II, etoposide, is approved for treatment of advanced NSCLC. Although typically used in combination with cisplatin, the response rate for single agent etoposide in untreated NSCLC patients is 23% [44], which is similar to the prevalence of TOP2A expression in NSCLC tumors (Figure 3B). Because increased expression of TOP2A in immunohistochemistry can be associated with increased response to etoposide in other tumor types, our studies suggest that expression of TOP2A could be used to guide the application of etoposide therapy [45].

PTTG1 is a homolog of yeast securin, which prevent separins from promoting sister chromatid separation [16]. PTTG1 has transforming activity *in vitro* and tumorigenic activity *in vivo*, and the gene is highly expressed in various tumors [46]. Its oncogenic effects may result from modulation of p53 function [47]. Overexpression of PTTG plays a role in the genesis and progression of NSCLC [48].

Survivin is overexpressed in most human malignancies and implicated in mitosis regulation and preservation of cell viability. Mitochondrial Survivin inhibits apoptosis and promotes tumorigenesis [49]. Its role during lung tumorigenesis is supported by clinical data showing that Survivin transcripts are a defining diagnostic marker for NSCLC [50], and that gene overexpression is almost always present in early-stage NSCLC [51]. Our observation that cytoplasmic Survivin is preferentially expressed in stage 1 specimens is consistent with this. Although nuclear Survivin is almost exclusively expressed in tumor tissues, no clinical associations were observed. Conflicting observations have been reported with survivin in that nuclear survivin can be associated with better [52] or worse survival in NSCLC [53].

Taken together, our studies show that a comprehensive array approach can be valuable to identify new targets in NSCLC. The identification of cytoplasmic loss of CDK1 in lung cancer as an independent poor prognostic factor in NSCLC patients is a surprising finding with potential clinical significance in that CDK1 may play an important role in chemotherapeutic responsiveness distinct from its role in cell cycle progression. Future studies will assess the utility of stratifying patients and optimizing chemotherapy in NSCLC protocols based on expression of TOP2A and cytoplasmic CDK1.

## Materials and Methods

### Cell lines and drugs

NHBE, BEAS-2B [12], NNK-BEAS-2B [13] and H157 cells were incubated at 37°C in 5% CO_2_. When cultures reached 90% confluence, cells were harvested and frozen at −80°C prior to RNA and protein isolation. NHBE cells were obtained from Cambrex Bio Science Walkersville, Inc. (Walkersville, MD), and were cultured using Bronchial Epithelial Growth Medium (BEGM) containing 2 µl/ml bovine pituitary extract, 5 µg/ml insulin, 0.5 µg/ml hydrocortisone, 0.5 ng/ml human epidermal growth factor, 0.5 µl/ml epinephrine, 10 µg/ml transferrin, 0.1 ng/ml retinoic acid, 6.5 ng/ml triiodothyronine, 50 µg/ml gentamycin and 50 ng/ml amphotericin-B. The BEAS-2B and NNK-BEAS-2B were maintained with LHC-8 (Biofluids, MD) with 100 units/ml penicillin and 100 µg/ml streptomycin (GIBCO-BRL, Gaithersburg, MD). The H157 lung squamous cell carcinoma cell line was provided by H. Oie and Dr. F. Kaye at the National Cancer Institute/Naval Medical Oncology (Bethesda, MD), were maintained in RPMI 1640 medium with 10% fetal bovine serum with 100 units/ml penicillin and 100 µg/ml streptomycin (GIBCO-BRL, Gaithersburg, MD). All the chemotherapy drugs were provided by NCI/Navy Medical Oncology.

### RNA and protein isolation

RNA and protein were isolated from the same sample using TRIzol reagent according to the manufacturer's instructions (Invitrogen, Carlsbad, CA). Briefly, 1 ml of TRIzol reagent was used per 10 cm^2^ flask to lyse cells by rapid up and down pipetting and subsequently incubated at room temperature for 5 min, then mixed with 2/10 volume chloroform by vigorous shaking for 15 sec. Phase separation was achieved by placing the samples at room temperature for 3 min, then centrifuging 3,500 rpm at 4°C for 15 min. The RNA from the top aqueous layer was removed to another 50 ml tube, slowly mixed with equal volume 70% ethanol, and then transferred to RNeasy column (Qiagen, Inc., Valencia, CA) for purification. The protein from the remainder layers was precipitated, washed and dissolved according to the manufacturer's instructions, and quantified by MicroBCA assay reagent kit (Pierce, Rockford, IL). RNA was eluted from column with RNase-free water, concentrated to >7 µg/µl by centrifugation on a MicroCon YM-30 filter (Millipore, Billerica, MA), and quantified using a UV/visible spectrophotometer Ultrospec3000 (Amersham Biosciences, Piscataway, NJ). The integrity of total RNA was verified by denaturing gel electrophoresis.

### cDNA Microarray analysis

The cDNA microarrays contained over 28,798 cDNA clones representing over 23,600 unique Unigene cluster and were constructed as described [54]. Gene's names are listed according to Unigene build 141 (www.ncbi.nlm.nih.gov/unigene). Fluorescently labeled cDNA were synthesized from 100 µg RNA by oligo(dT)-primed reverse transcription in the presence of Cy3- or Cy5-dUTP (Amersham Bisciences, Piscataway, NJ), as described [55], (http://www.nhgri.nih.gov/UACORE/index.html). Purified Cy3/Cy5-labelled probes were combined and hybridized in the presence of 2×Denhart's solution, 3.2×saline sodium citrate (SSC), and 0.5% sodium dodecyl sulfate (SDS) in a humidified chamber at 65°C overnight. Prior to scanning (Agilent Technologies, Foster City, CA), slides were successively washed at 22°C in 0.5×SSC/0.1% SDS for 2 min, 0.5×SSC/0.01% SDS for 2 min, and 0.06×SSC for 2 min. Image analyses were performed with the IPLab software (Fairfax, VA). The reference cell (NHBE) was included in the every individual hybridization to allow for normalization of each clone's expression relative to the reference for each cell line (BEAS-2B, NNK-BEAS-2B or H157). A self-to-self hybridization with dye reversal was performed to exclude preferential differences in probe labeling. Every sample was labeled with Cy5 and Cy3 and hybridized twice. The two fluorescent images (red and green channels) obtained from the scanner constituted the intensity raw data from which differential gene expression ratio and quality control values were calculated. All data were entered into a relational database, using the FileMaker Pro 5 software (Santa Clara, CA). Genes were identified as differentially regulated only if corrected red/green hybridization signals differed by at least twofold. The genes in each group were alphabetically listed and the ratios of BEAS-2B/NHBE, NNK-BEAS-2B/NHBE or H157/NHBE were graphically visualized by Cluster and TreeView programs (http://rana.lbl.gov/EisenSoftware.htm). These methods fulfilled the MIAME criteria (http://www.mged.org/miame). The raw data were deposited in a public functional genomics data repository Gene Expression Omnibus (http://www.ncbi.nlm.nih.gov/geo/), and the GEO accession number is GSE28282.

### High-Throughput GoMiner analysis

To integrate information and illuminate patterns from multiple microarrays in relationship to the Gene Ontology, High-Throughput GoMiner (http://discover.nci.nih.gov/gominer/htgm.jsp) analysis was performed following the instructions [56].

### Immunoblotting

50 µg cellular proteins precipitated from NHBE, BEAS-2B, NNK-BEAS-2B and H157 cells lysates were analyzed by immunoblotting after SDS-PAGE electrophoresis. Individual primary antibodies used were rabbit polyclonal antibodies that recognized CDK1, phospho-CDK1(Tyr15), FKHR, STMN1 (Cell signaling, MA), PLK1 (Upstate, Lake Placid, NY), Cyr61, ETS1, p15, GKLF/KLF4, phospho-Thr34 Survivin (Santa Cruz Biotechnology, Santa Cruz, CA), PERP, TRAIL (Poway, CA), goat polyclonal antibodies that recognized PTTG1, PBK, hnRNP A2/B1, galactin1, DNMT1, cyclin B2, HIP1, SPARC, S100A8, or mouse monoclonal antibodies that recognized TOP2A (Roche, IN), Survivin (Cell signaling, MA), maspin, TAO1 (BD Bioscience, San Jose, CA), cyclin A, p63 (Santa Cruz Biotechnology, Santa Cruz, CA) and KRT 14 (USBiological, Swampscott, MA). Rabbit anti-human XAGE-1 polyclonal antibody was provided by Dr. Ira Pastan (NCI). Immunocomplexes were visualized by SuperSignal West Pico Chemiluminescent Substrate (Pierce, Rockford, IL) using goat anti-rabbit or mouse (Cell signaling, MA) or rabbit anti-goat (Santa Cruz Biotechnology, Santa Cruz, CA) IgG coupled to horseradish peroxidase as a secondary antibody.

### Lung tissue microarray and immunohistochemical staining

Three hundred lung cancer cases (150 pulmonary AD and 150 SCC cases) were selected from the Armed Forces Institute of Pathology archive, and demographic and clinical data were collected at the time of the paraffin block acquisition. A total of 246 patients had complete clinical information. Survival time and outcome data were available for 211 patients. Approval for use of the tissue in these research studies was obtained from both the Institutional Review Boards of the Armed Forces Institute of Pathology and the Office of Human Subjects Research of the National Institutes of Health (Bethesda, MD). Approval for use of the tissues in these studies was obtained from both the Institutional Review Boards of the Armed Forces Institute of Pathology and the Office of Human Subjects Research of the National Institutes of Health (Bethesda, MD), who waived the need for consent since the specimens were anonymized. The tumor were staged according to the International Union against Cancer's tumor-node-metastasis (TNM) classification and histologically subtyped and graded according to WHO guidelines. The most representative tumor areas were carefully selected based on the matched H&E-stained slides and marked directly on the donor block. One hundred adjacent non-diseased lung tissues from AD and SCC cases, along with 50 non-pulmonary normal tissues, were included in the same array block. Immunochemical staining was carried out on this tissue microarray using a standard avidin-biotin-perioxidase complex technique (Vectastain Elite ABC kit; Vector Laboratory). The optimized titer and specificity were determined using lung sausage slides (FGMULTI-7; BioGenex) for each primary antibody. The primary antibodies are mouse monoclonal (A17) anti-p34 Cdc2 (CDK1) (ab18; abcam), mouse monoclonal (KiS1) anti-topoisomerase II-alpha (TOP2A) (Roche), rabbit polyclonal anti-human Pituitary Tumor-Transforming Gene (PTTG1) (Zymed Laboratories Inc.) and mouse monoclonal (D-8) anti-Survivin (sc-17779; Santa Cruz Biotech) antibodies, and at dilutions of 1∶125, 1∶100, 1∶50 and 1∶10, respectively. The optimized staining condition for lung tumor microarray was determined based on the coexistence of both positive and negative cells in the same tissue sample. The criteria for the staining were scored as follows: distribution score was scored as 0 (0%), 1 (1–50%; 1–10% for Survivin nuclear staining) and 2 (51–100%; 11–100% for Survivin nuclear staining) to indicate the percentage of positive cells in all tumor cells present in one tissue. The intensity score was scored as 0 (no signal), 1 (weak), 2 (moderate) and 3 (marked). The total of distribution score and intensity score was then summed into a total score (TS) of TS0 (sum = 0), TS1 (sum = 2), TS2 (sum = 3), and TS3 (sum = 4–5). TS0 or TS1 was regarded as negative, and TS2 or TS3 was regarded as positive.

### Degradation and inactivation of CDK1 from cytoplasm in FT210 Cells

The murine carcinoma cell lines FM3A and FT210 [15] were maintained in RPMI supplemented with fetal bovine serum (10%, v/v) at 32°C. FT210 is a temperature-sensitive p34cdc2/Cdk1 mutant cell line derived from FM3A. For reducing the level and activity of Cdk1, FT210 cells were cultured at the non-permissive temperature of 39°C. FT210 cells were cultured at the permissive temperature of 32°C as control. Its parent wild-type FM3A cells were treated in the same way for comparison. For preparation of cytoplasmic and nuclear extracts, 10×10^6^ cells were harvested, washed and pelleted, then resuspended in 100 µl Lysis Buffer (10 mM Tris⋅HCl, pH 8.0; 60 mM KCl; 1 mM EDTA; 0.5% NP-40; 1 mM DTT, 0.2 mM PMSF and Protease Inhibitors cocktail were freshly added). Incubated 5 min on ice, then centrifuged 2300 rpm for 5 min at 4°C. Transferred and stored the supernatant at −80°C. Added 40 µl of Nuclear Extract Buffer (20 mM Tris⋅HCl, pH 8.0; 420 mM NaCl; 0.2 mM EDTA; 25% glycerol; 1.5 mM MgCl_2_; 1 mM DTT, 0.2 mM PMSF and Protease Inhibitors cocktail were freshly added) to the nuclear pellet, and incubated on ice for 10 min. Centrifuge at 14,000 rpm for 5 min at 4°C, transferred and stored nuclear extracts at −80°C. Then cytoplasmic and nuclear extracts were subjected to immunoblotting analysis. CDK1 activity was monitored by detecting the phosphorylation of Survivin on Thr34, a site for CyclinB-CDK1 kinase, with p-Survivin (Thr34) antibody (Santa Cruz Biotechnology, Inc.).

### Pharmacologic treatments and apoptosis assay

To test chemotherapeutical response in Cdk1 degradation and inactivation situations, FT210 or FM3A cells were cultured at 32°C or 39°C for 1–4 days by adding etoposide (10 µM), cisplatin (10 µM), paclitaxel (1 µM), docetaxel (1 µM) and vinorelbine (0.1 µM). In etoposide and cisplatin treatments, the cells were serum-starvated in 0.1% FBS medium overnight and treated in 0.1% FBS containing medium. Normal medium was used in all microtubule-targeting reagents treatments. For CDK1 overexpression in H157 cells experiments, added the paclitaxel (1 µM) at post-transfection 48 h and treated for another 48 h. In CDK1 knockdown in H157 and A549 cells experiments, added the same concentration of paclitaxel at post-transfection 72 h and treated for 48 h. The CDK1 overexpression or underexpression by transfecting *CDK1* plasmid or siRNA in H157 and A549 cells were confirmed by immunoblotting, and related antibodies such as p42MAPK, CDK1, Cleaved PARP and Wee1 were from Cell Signaling Technology. For apoptosis analysis, floating cells and adherent cells were harvested by trypsinization and then centrifuged at 1000×*g* for 5 min. Cells were fixed in ice-cold 70% methanol added dropwise and then incubated at −20°C for 30 min. Cells were centrifuged and incubated with propidium iodide (25 µg/ml) supplemented with RNaseA (30 µg/ml) for 30 min at room temperature. Quantification of sub-G1 DNA content was determined by flow cytometry analysis using a Becton Dickinson FACSort and by manual gating using CellQuest software. Apoptotosis experiments were performed in triplicate and were independently repeated at least three times. The results represent mean ± SE from three independent experiments. The difference of the comparison was tested using T-test with two samples assuming equal variance, and p<0.001 was set as significant.

### Transfection of DNA constructs or siRNA duplexes

Plasmid DNA and siRNA duplex were delivered into the cells by nucleofection technology (Lonza). Lung cancer cell lines H157 or A549 cells were maintained in RPMI1640+10% FBS and harvested for nucleofection when reaching a 70∼80 confluency. The number of cells per nucleofection is 2×10^6^. The nucleofection was performed using the nucleofector kit V with T-16 nucleofection program according to the manufacturer's instructions. The transfection efficiency was monitored by fluorescence using pmaxGFP (about 98% for H157 and 80% for A549 positive rates based on flow cytometry and microscopy analyses). 2 µg plasmid constructs or siRNA double stranded RNA duplexes (16 µl 10 µM siRNA) were used for each nucleofection. *CDK1* wild type (pCMV-*cdc2WT*) and dominant negative (pCMV-*cdc2D146N*) constructs were provided by Drs. Sander van den Heuvel and Ed Harlow (Massachusetts General Hospital). *CDK1* and control siRNAs were from Cell Signaling Technology.

### NCI60 human cancer cell line COMPARE analysis

National Cancer Institute drug-screening panel of 60 human cancer cells (NCI60) includes lung, colon, breast, ovarian, leukemia, renal, melanoma, prostate and CNS cancer cells. To ascertain the relevance of CDK1 to chemotherapeutic sensitivity in a broad range, we performed COMPARE analysis. We used paclitaxel (NSC125973) as a seed, 50% growth inhibition (GI_50_) and 2-days as assay endpoint and time to search molecular targets in NCI60 panel, and found CDK1 activator CDC25A, p-Y15/T14 CDK1, CDK1, and CDK1 partners cyclin A/B were positive associated paclitaxel drug sensitivity on the top of rank-order list. We further used all the agents (vinorelbine/navelbine, NSC 608210, NCI60 set data is not available) we used in the experiments and the above molecular targets to run MATRIX COMPARE (http://itbwork.nci.nih.gov/PublicServer/jsp/Form_Standard.jsp). Every compound has been done several times with different cell line numbers (the highest log of GI_50_ concentration in every assay is different), and every molecular target has primary and log two forms, therefore there are a lot of combinations between compounds and targets. Because the Pearson Correlation Coefficients (PCC) of the different combinations between individual compound and target are similar, only one assay from each compound and only log form (because the mean graph of drug sensitivity is expressed as the logarithm of the concentration resulting in 50% growth inhibition) of Western blot densitometry from each targets were listed. The PCC was ranked on protein target list when it was not less than 0.200. Other protein targets were listed if their PCC have higher ranks than any ranked one of the above targets.

### Statistical analysis

Comparisons of clinical and pathological factors between positive and negative groups were performed using the χ^2^ test. The log-rank test was used for comparing survival distribution between positive and negative groups of staining, and Kaplan-Meier curves were plotted for the two groups. Association of binary staining status between each pair of antibodies was examined using the χ^2^ test, and the association was considered significant if *p*≤0.01. Association between antibody staining and clinical factors was also analyzed using the χ^2^ test, where *p*≤0.05 was considered significant.

## Supporting Information

Figure S1 **Gene expression changes in cell cycle and checkpoint controls.** DNA replication occurs in S phase and chromosomes segregation into daughter progeny occurs in mitosis (M phase). G1 and G2 are DNA synthesis and mitosis preparation phases, respectively. In normal cells, cell cycle arrest occurs most frequently at the G1/S and G2/M boundaries. Changes in gene expression from groups A and J would be predicted to affect every phase of the cell cycle. For example, increased expression of *PCNA*, *MCM7*, *Cyclin E2/A*, *CDK1*, *TPX2* and *MAD2L1*, and decreased expression of *14-3-3σ* and *CHK2* would be expected to promote G1 to S and G2 to M transitions, as well as escape from DNA damage-induced arrest and cell cycle checkpoint control. Upregulation of *MCM3* and *SMAD6* are associated with SV40-mediated immortalization but are lost in fully transformed NNK-BEAS-2B cells. Upregulation of *ASK*, *CDC3A*, *Cyclin B2*, *PBK*, *14-3-3ε/η*, *BUB1* and *BUB1B*, as well as downregulation of *p21Cip1*, is characteristic of both types of transformed cells, indicating that the dysregulation of cell cycle from S phase to mitosis might be more affected in the process from immortalization to full transformation. NNK-specific genes also encompassed the entire cell cycle because overexpression of *CDK4/2*, *CDC45/25B/20*, *CyclinG1/F*, *HDAC* family members, *E2F* family members, *DP1*, *STK15*, *Survivin* and *PLK1*, and underexpression of *GSK3β*, *FKHR*, *E2F6* and *GADD45γ* were observed. The cumulative effect of these changes in gene expression would be predicted to increase autonomous progression through the cell cycle.
(TIF)

Figure S2 **Gene expression changes in apoptosis pathways.** Apoptosis can be initiated through either death receptors on the cell surface (extrinsic) or mitochondria (intrinsic) pathways. Induction of apoptosis leads to activation of the initiator caspase-8 and 10 (extrinsic) or 9 (intrinsic), which can activate executioner caspases to cleave the death substrates and eventually results in apoptosis. There are crosstalks between these two pathways. Changes in gene expression during transformation also would be predicted to alter the balance between apoptosis and cellular survival. Apoptosis promoting factors such as *TRAIL* and *PERP* were underexpressed in immortalized and transformed cells. NNK-specific changes included increased expression of anti-apoptotic factors *DCR3*, *cIAP2*, *Survivin* and *BCL-X* and decreased expression of pro-apoptotic factors *Granzyme B*, *TNFR1*, *TRAF1*, *C-Jun*, *APAF-1* and *Caspase-1*. These NNK-induced alterations in gene expression favored an anti-apoptotic trend that might contribute to lung tumorigenesis.
(TIF)

Table S1 **Classification of 1,688 regulated genes among BEAS-2B(B), NNK-BEAS-2B(NB) and H157(H) compared to NHBE(N).**
(XLS)

Table S2 **The genes involved in significantly changed GeneOntology categories in BEAS-2B, NNK-BEAS-2B and H157 compared to NHBE cells.**
(XLS)

Table S3 **The NNK-specific groups' GeneOntology categories and related genes.**
(XLS)
