# Supplementary figures and images for: Loss of Cytoplasmic CDK1 Predicts Poor Survival in Human Lung Cancer and Confers Chemotherapeutic Resistance
Source: PLoS One. 2011 Aug 24;6(8):e23849. doi: 10.1371/journal.pone.0023849 (PMC3161069; doi:10.1371/journal.pone.0023849)

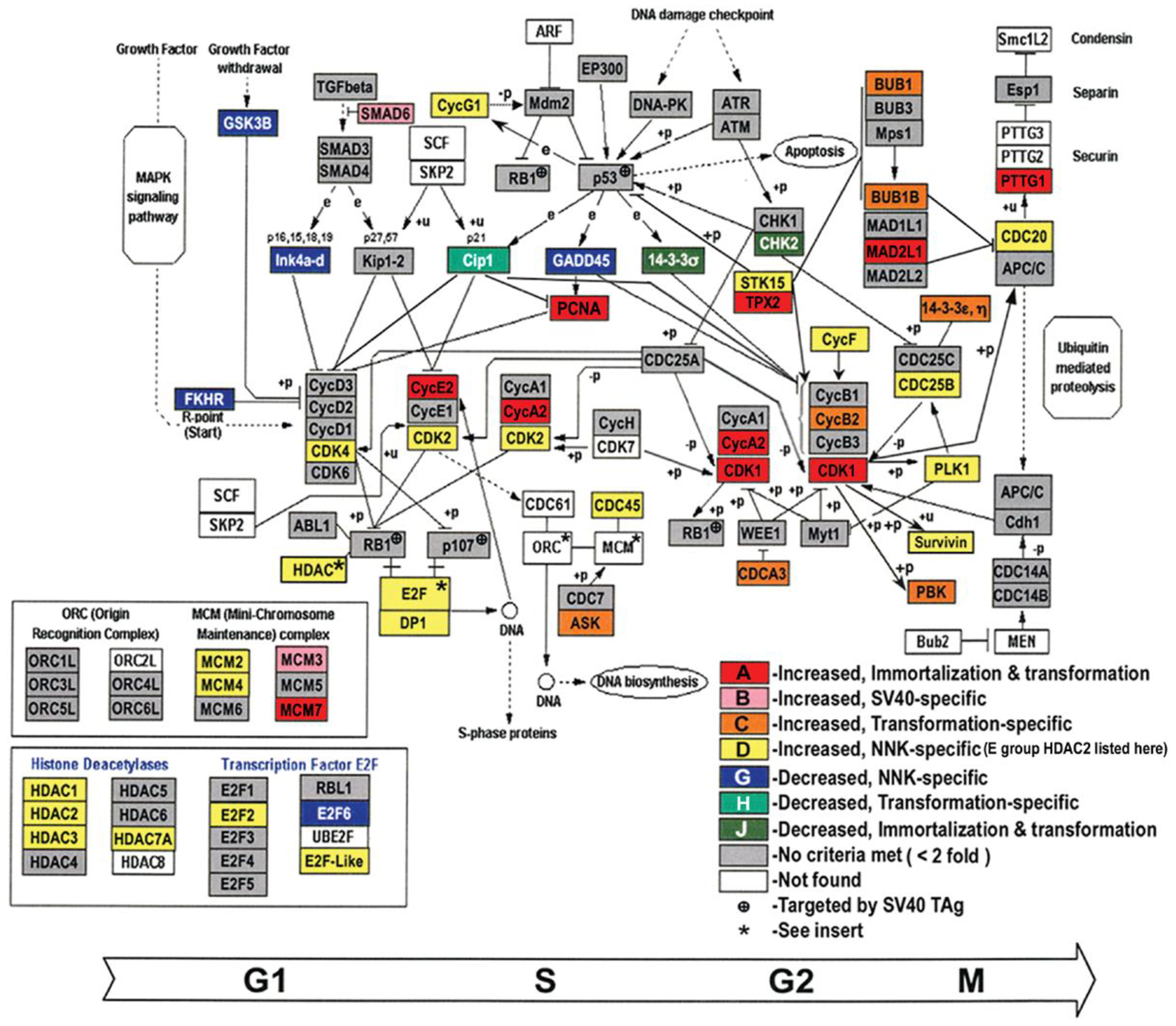

Supplement: Figure S1 — Gene expression changes in cell cycle and checkpoint controls. DNA replication occurs in S phase and chromosomes segregation into daughter progeny occurs in mitosis (M phase). G1 and G2 are DNA synthesis and mitosis preparation phases, respectively. In normal cells, cell cycle arrest occurs most frequently at the G1/S and G2/M boundaries. Changes in gene expression from groups A and J would be predicted to affect every phase of the cell cycle. For example, increased expression of PCNA, MCM7, Cyclin E2/A, CDK1, TPX2 and MAD2L1, and decreased expression of 14-3-3σ and CHK2 would be expected to promote G1 to S and G2 to M transitions, as well as escape from DNA damage-induced arrest and cell cycle checkpoint control. Upregulation of MCM3 and SMAD6 are associated with SV40-mediated immortalization but are lost in fully transformed NNK-BEAS-2B cells. Upregulation of ASK, CDC3A, Cyclin B2, PBK, 14-3-3ε/η, BUB1 and BUB1B, as well as downregulation of p21Cip1, is characteristic of both types of transformed cells, indicating that the dysregulation of cell cycle from S phase to mitosis might be more affected in the process from immortalization to full transformation. NNK-specific genes also encompassed the entire cell cycle because overexpression of CDK4/2, CDC45/25B/20, CyclinG1/F, HDAC family members, E2F family members, DP1, STK15, Survivin and PLK1, and underexpression of GSK3β, FKHR, E2F6 and GADD45γ were observed. The cumulative effect of these changes in gene expression would be predicted to increase autonomous progression through the cell cycle. (TIF) [file pone.0023849.s001.tif]

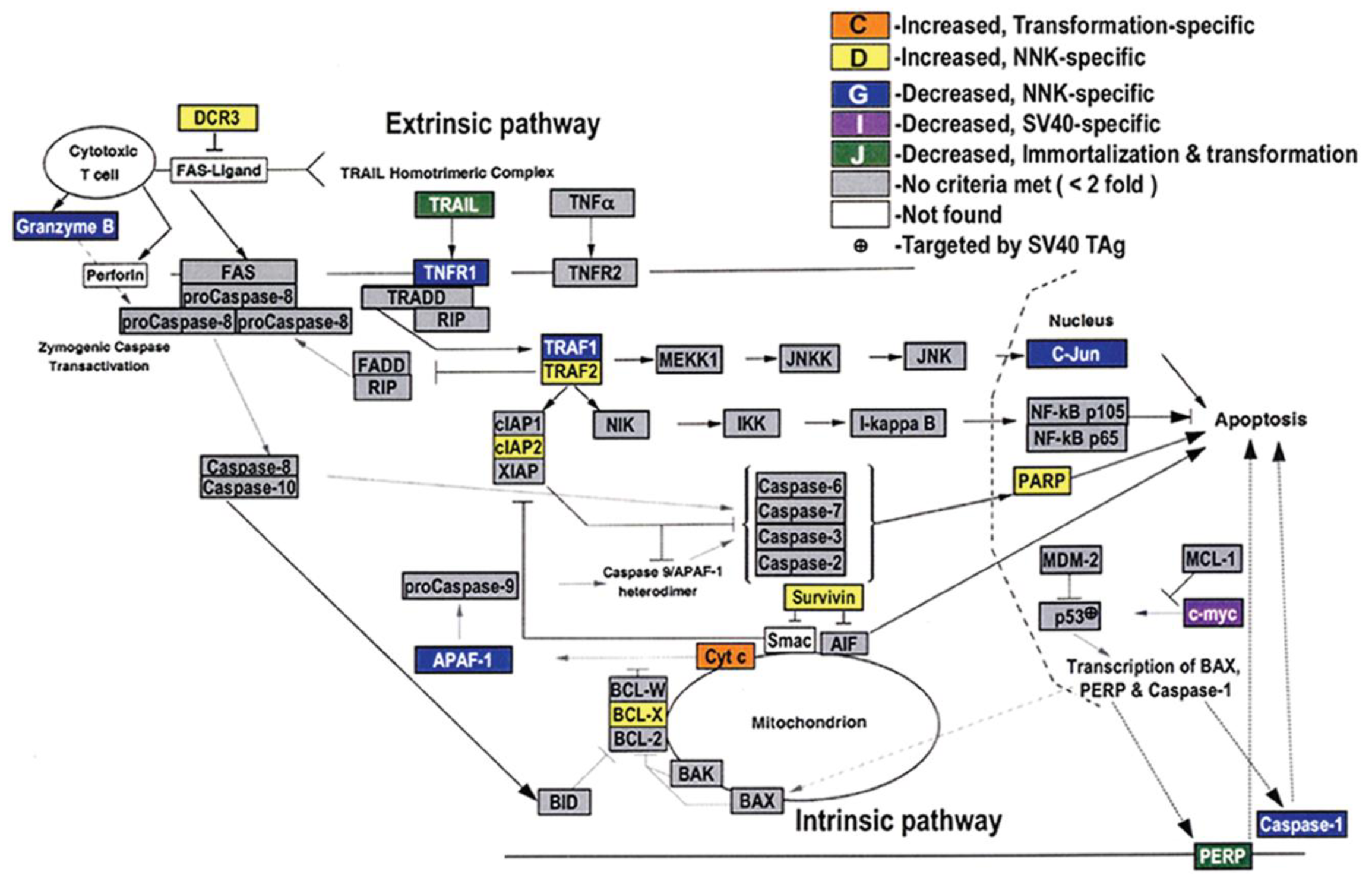

Supplement: Figure S2 — Gene expression changes in apoptosis pathways. Apoptosis can be initiated through either death receptors on the cell surface (extrinsic) or mitochondria (intrinsic) pathways. Induction of apoptosis leads to activation of the initiator caspase-8 and 10 (extrinsic) or 9 (intrinsic), which can activate executioner caspases to cleave the death substrates and eventually results in apoptosis. There are crosstalks between these two pathways. Changes in gene expression during transformation also would be predicted to alter the balance between apoptosis and cellular survival. Apoptosis promoting factors such as TRAIL and PERP were underexpressed in immortalized and transformed cells. NNK-specific changes included increased expression of anti-apoptotic factors DCR3, cIAP2, Survivin and BCL-X and decreased expression of pro-apoptotic factors Granzyme B, TNFR1, TRAF1, C-Jun, APAF-1 and Caspase-1. These NNK-induced alterations in gene expression favored an anti-apoptotic trend that might contribute to lung tumorigenesis. (TIF) [file pone.0023849.s002.tif]
